# The glycoconjugate ontology (GlycoCoO) for standardizing the annotation of glycoconjugate data and its application

**DOI:** 10.1093/glycob/cwab013

**Published:** 2021-02-23

**Authors:** Issaku Yamada, Matthew P Campbell, Nathan Edwards, Leyla Jael Castro, Frederique Lisacek, Julien Mariethoz, Tamiko Ono, Rene Ranzinger, Daisuke Shinmachi, Kiyoko F Aoki-Kinoshita

**Affiliations:** Research Department, The Noguchi Institute, 1-9-7 Kaga, Itabashi, Tokyo 173-0003, Japan; Institute for Glycomics, Griffith University at Gold Coast, Southport, QLD 4215, Australia; Department of Biochemistry, Molecular and Cellular Biology, Georgetown University Medical Center, Washington, D.C. 20007, USA; ZB MED Information Centre for Life Sciences, Gleueler Str. 60, 50931 Cologne, Germany; Proteome Informatics Group, SIB Swiss Institute of Bioinformatics, Computer Science Department, University of Geneva, route de Drize 7, CH – 1227 Geneva Switzerland, and also Section of Biology, University of Geneva, Geneva, Switzerland; Proteome Informatics Group, SIB Swiss Institute of Bioinformatics, 7 Route de Drize, 1227 Geneva, Switzerland; Faculty of Science and Engineering, Soka University, 1-236 Tangi-machi, Hachioji, Tokyo 192-8577, Japan; Complex Carbohydrate Research Center, The University of Georgia, 315 Riverbend Rd, Athens, Georgia 30602, USA; R&D Department, SparqLite LLC., 1615-22 Ishikawamachi, Hachioji, Tokyo 192-0032, Japan; Glycan & Life Science Integration Center (GaLSIC), Faculty of Science and Engineering, Soka University, 1-236 Tangi-machi, Hachioji, Tokyo 192-8577, Japan

**Keywords:** glycoconjugate, glycolipid, glycoprotein, ontology, Semantic Web

## Abstract

Recent years have seen great advances in the development of glycoproteomics protocols and methods resulting in a sustainable increase in the reporting proteins, their attached glycans and glycosylation sites. However, only very few of these reports find their way into databases or data repositories. One of the major reasons is the absence of digital standard to represent glycoproteins and the challenging annotations with glycans. Depending on the experimental method, such a standard must be able to represent glycans as complete structures or as compositions, store not just single glycans but also represent glycoforms on a specific glycosylation side, deal with partially missing site information if no site mapping was performed, and store abundances or ratios of glycans within a glycoform of a specific site. To support the above, we have developed the GlycoConjugate Ontology (GlycoCoO) as a standard semantic framework to describe and represent glycoproteomics data. GlycoCoO can be used to represent glycoproteomics data in triplestores and can serve as a basis for data exchange formats. The ontology, database providers and supporting documentation are available online (https://github.com/glycoinfo/GlycoCoO).

## Introduction

Glycobiology is the study of saccharides (also called carbohydrates, sugar chains or glycans) that are widely distributed in nature. The importance of glycobiology can be understood by considering the fact that they encompass some of the major posttranslational modifications of proteins, as carbohydrates help explain how the relatively small number of genes in the typical genome can generate the enormous biological complexities inherent in the development, growth and functioning of diverse organisms (Varki and Kornfeld 2017).

The biological roles of carbohydrates are particularly prominent in the assembly of complex multicellular organs and organisms, which requires interactions between cells and the surrounding matrix. Without any known exception, all cells and numerous macromolecules in nature carry a repertoire of covalently attached glycans (albeit glycans can also be freestanding entities). Glycoproteins are frequently located on the cell membrane or secreted; therefore, modulating or mediating a variety of events in cell–cell, cell–matrix and cell–molecule interactions critical to the development and function of a complex multicellular organism including cellular activation, embryonic development, differentiation and malignancy. They can also mediate interactions between organisms (e.g., between host and a parasite, pathogen or a symbiont). Consequently, understanding the roles of glycans, changes in glycoforms/abundance of glycans, and site-occupancy are essential for improving our understanding of cellular systems. In the last few years improvements to bioinformatics tools and databases including data standardization and interoperability have helped glycobiologists better understand their functions.

Over the last few decades several initiatives have cataloged and organized glycan-related information in databases. These activities started with CarbBank a database project for glycan structures which was initiated in 1987 but ceased operation in 1997 due to lack of funding support (Doubet and Albersheim 1992). The final version of the database contained ∼50,000 records comprising over 23,000 glycan sequences with associated biological background, experimental method and publication information. This data set has been used to seed new databases with a basic set of glycan structure records by follow up database projects, including the Kyoto Encyclopedia of Genes and Genomes (KEGG) Glycan (Kanehisa 2017), the database of the US Consortium for Functional Glycomics (CFG) (Raman et al. 2006), GLYCOSCIENCES.de (Lütteke et al. 2006), GlycoSuiteDB (Cooper et al. 2003), UniCarbKB (Campbell et al. 2017), Carbohydrate Structure Database (CSDB) (Egorova and Toukach 2016), GlycomeDB (Ranzinger et al. 2011) and EUROCarbDB (von der Lieth et al. 2011).

In brief, KEGG Glycan is an integrated knowledge base of protein networks with genomic and chemical information and provides access to glycan structures through the manually drawn pathway maps representing the current knowledge of glycan biosynthesis and metabolism for various species. EUROCarbDB established the technical requirements for developing a centralized and standardized database architecture for carbohydrate-related structure data and analytical data from liquid chromatography, mass spectrometry and nuclear magnetic resonance (NMR) experiments. Several resources were developed under EUROCarbDB including, MonosaccharideDB (Lütteke 2017), and the separation-focused database GlycoBase (Campbell et al. (2015)) that was later migrated to GlycoStore (Zhao et al. 2018). GLYCOSCIENCES.de imported the entire CarbBank dataset and focuses on the three-dimensional conformations of carbohydrates as extracted from PDB and has been recently updated with Glycosciences.DB (Böhm et al. 2019). The CFG database integrates human and mouse tissue and cell line glycan mass spectrometry profiling and glycan microarray binding data produced by the consortium members. Recently, the CFG transitioned to the NCFG with a focus on advancing glycan microarray technologies with supporting informatics.

More recent developments include the CSDB, which stores structural, bibliographic, taxonomic, NMR spectroscopic and other data on natural carbohydrates and their derivatives comprising the Bacterial CSDB and the Plant/Fungal CSDB (Toukach and Egorova 2016). UniCarb-DB (Hayes et al. 2011) stores glycan structures with corresponding experimental mass spectra while UniCarbKB and GlyConnect (Alocci et al. 2019) are extensions of GlycoSuiteDB, a mammalian glycoprotein centric database that provides structure and site-specific glycoprotein information curated from the literature. Between 2011 and 2016, GlycomeDB served as a centralized resource for storing glycan structures reported in almost all publicly available glycan structure databases. It merged with GlyTouCan (Fujita et al. 2021) in an international collaboration to provide a repository for depositing glycan structures, compositions and topologies, with each entry assigned a unique accession number. Out of the above-mentioned databases only GlycoSuiteDB and its successors UniCarbKB and GlyConnect store carbohydrate structures and glycoproteomics information (e.g., which protein the glycan was attached to and specific position); UniCarbKB data collections are being integrated with GlyGen (York et al. 2020).

Semantic Web technologies, which involve the development of ontologies, controlled vocabularies and Resource Description Framework (RDF) data available from SPARQL endpoints, enables efficient integration of disparate data resources (Aoki-Kinoshita et al. 2015; Katayama et al. 2014; Aoki-Kinoshita et al. 2013). We have shown that compared to traditional Relational Database Management Systems (RDBMS), RDF allows dynamic queries to be made across resources simultaneously. This was demonstrated by the development and adoption of an ontology for glycan structures, called GlycoRDF (Ranzinger et al. 2015). To further substantiate our choice of RDF, we compared modeling glycan data with Neo4J graph database and demonstrated the advantages of the latter (Alocci et al. 2015). Albeit there is a bottleneck where designing the most appropriate queries may be difficult, many solutions are being developed to allow users to use natural language that can be translated to SPARQL (Ferré 2016; Damljanovic et al. 2012; Song et al. 2019; McCarthy et al. 2012; Barrière 2016; Chiba and Uchiyama 2017).

GlycoRDF was a first step to integrate glycan data across disparate databases. Glycan structures are now linked across various databases by GlyTouCan which has also be implementing Semantic Web technologies by utilizing GlycoRDF. However, glycans function together with other molecules such as proteins and lipids, forming glycoconjugates, which is a term used for glycans that are linked to proteins or lipids, otherwise known as glycoproteins or glycolipids, respectively. With the progress of glycoscience research, studies targeting glycoconjugates have accelerated, and various research results have been reported in the literature.

The adoption of GlycoRDF by various databases including GlyTouCan, UniCarbKB, and CSDB, has improved data interoperability in the glycosciences and made it clear that an ontology for glycoconjugates was needed. Several lipid databases exist which contain glycolipids in part, including LIPID MAPS (Sud et al. 2007), LipidBank (Watanabe et al. 2000) and SwissLipids (Aimo et al. 2015). UniProt (The UniProt Consortium 2017) and NeXtProt provide information on site-specific protein glycosylation and serve as major sources of information. Recently, several projects have started to integrate glycomics, glycoproteomics and glycolipidomics data. Such diversity and information rich data collections require a solid framework for representing and sharing glycoconjugate information in a standardized way.

Here we present a glycoconjugate ontology, named GlycoCoO, for describing glycoconjugate structures and their functions, an ontology which will promote integration of data within the related fields of glycoscience, protein and lipid sciences. GlycoCoO can express not only the chemical structural information of a glycoconjugate but also its linked data and annotation such as glycan abundance ratio, disease, bibliographic information, sample information, etc. By integrating data constructed using GlycoCoO through Semantic Web technology, not only can life science researchers improve convenience when using these databases, but also more users across other fields can be expected to take advantage of this information. The role of data science is expected to become more important in life science research. The interest of many researchers in converting research results into data can be expected to help the development of the field.

## Methods

### Ontology development

**Fig. 1:**
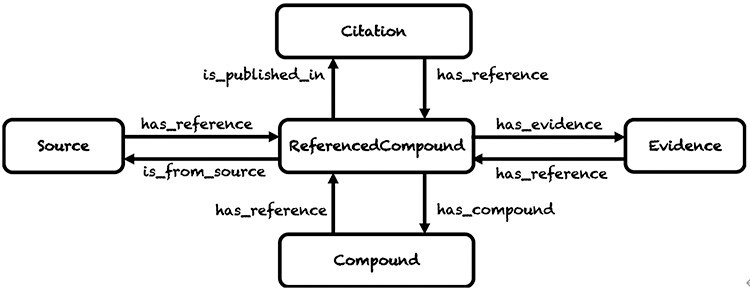
Overview of the GlycoRDF ontology, which defines a ReferencedCompound class that instantiates a Compound with its evidence, source and citation information.

GlycoRDF was originally developed to encapsulate metadata that most pertained to glycan structures. This included publications, the sample from which the glycan was obtained (biological or synthesized) and the experimental method used to obtain or analyze the glycan (e.g., mass spectrometry (MS), lectin binding, or nuclear magnetic resonance (NMR)). Because the same glycan could be found using different means and published in different papers, a new concept of “ReferencedCompound” was created to keep sets of these metadata independent from one another for the same glycan (see Figure Fig. 1). In this figure, a Compound is the superclass of Glycan, which would normally point to a GlyTouCan ID or similar. For a particular instance of a glycan, a ReferencedCompound would be created and linked with its related data including citation, experimental evidence and source information.

**Fig. 2:**
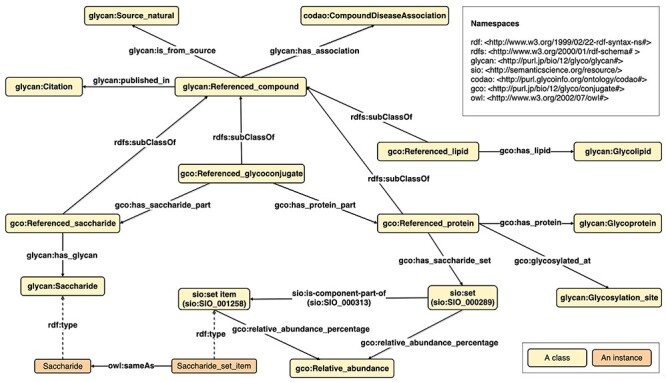
The GlycoCoO RDF schema for representing glycoconjugates, including glycoproteins and glycolipids.

**Fig. 3:**
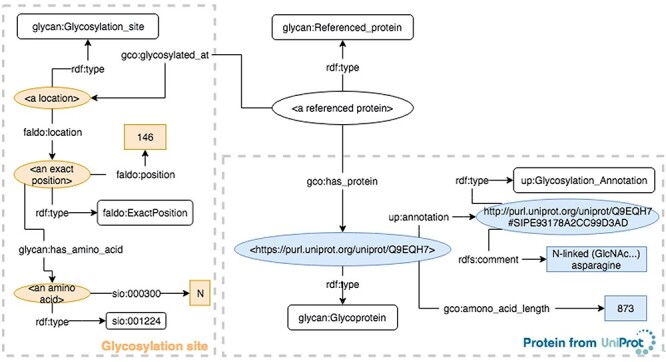
An example of a glycoprotein illustrated based on the GlycoCoO ontology. UniProt entry Q9EQH7 is a glycoprotein with six glycosylation sites. The example shows the representation of the site at location 146, which is an asparagine.

Since we wanted to reuse the GlycoRDF ontology to represent glycans in GlycoCoO, subclasses of ReferencedCompound were created, including ReferencedGlycoconjugate, ReferencedProtein and ReferencedLipid. By making these subclasses of “ReferencedCompound,” it became possible to describe the relationship of these biomolecules with their related metadata such as disease, publications and species using the same mechanism already implemented in GlycoRDF. Figure Fig. 2 illustrates the GlycoCoO schema, and Figure Fig. 3 is an example of a glycoprotein using this schema.

## Results

GlycoCoO makes it easier to integrate data from other resources. Following the ontology definition as described above three databases containing glycoconjugate data have implemented this ontology to represent their respective datasets. Each of these databases and their available RDFized datasets are as follows:

### UniCarbKB

UniCarbKB is a mammalian glycoprotein centric database that provides access to curated site-specific and global N- and O-glycosylation data. It expands on GlycoSuiteDB and EUROCarbDB with data curated from an additional 80 publications. Although UniCarbKB provides annotated entries for all species, its primary focus is the annotation of glycoproteins from mammalian systems of distinct taxonomic groups. For each glycoprotein record, two levels of annotation are provided where known: (i) data that denotes glycan structures characterized for a single purified glycoprotein with knowledge of the site of the glycosylation and (ii) site-specific data describing the glycan structures at specific sites of the protein. For site-specific annotations the UniCarbKB SPARQL endpoint (http://sparql.unicarbkb.org) provides access to approximately 1530 glycoprotein entries with over 4000 annotated glycosylation sites, and 4000 glycan structures (partial and fully defined). UniCarbKB also provides information on the biological source (taxonomy and tissue as described by NCBI MeSH (ROGERS 1963) and Uberon (Haendel et al. 2014)), disease state using the Disease Ontology (Schriml et al. 2012), and experimental methods and keywords (Campbell and Packer 2016). For updates and documentation refer to https://unicarbkb-glycostore.gitbook.io/data/.

### GlyConnect

GlyConnect is a glycoprotein and glycopeptide database providing curated experimental glycosylation data and the related contextual information like taxonomy, expression tissue or disease state. The dataset is built with 22,600 glycosylation sites on roughly 2,200 UniProtKB referenced glycoproteins, almost 4,000 glycans and 3,400 glycosylation sites. The curated data is supported by 900 articles. This collection includes several large-scale glycoproteomics studies that span 3,300 human N- and O-glycopeptides. It also makes references to biological context using Uberon (Mungall et al. 2012), Cell Ontology (Diehl et al. 2016), Gene Ontology (Gene Ontology Consortium 2015), Cellosaurus (Bairoch 2018) and Disease Ontology. The GlyConnect SPARQL endpoint (https://glyconnect.expasy.org/rdf) is being prepared and will be release by the end of 2019.

### GlycoNAVI

GlycoNAVI (https://glyconavi.org) is a web portal providing tools and datasets for glycoscientists. The GlycoAbun dataset of GlycoNAVI (https://glyconavi.org/GlycoAbun/) stores information of glycan abundance ratios of glycoforms on glycoconjugates. This dataset was manually curated from the literature and is also integrated in the GlyCosmos project. The GlycoNAVI SPARQL endpoint (https://sparql.glyconavi.org/sparql) provides to access to 1,297 glycans, 178 abundance ratio data, 102 disease states, 9 tissues and 178 articles.

As a proof of concept, the RDF data for a glycoprotein (UniProt ID: P00738) was extracted from all three major glycoprotein data resources (UniCarbKB, GlyConnect@ExPASy and GlycoNAVI) containing metadata from their respective resources. All of these data files are available on the GlycoCoO GitHub Wiki under RDF_Sample (https://github.com/glycoinfo/GlycoCoO/tree/master/RDF_Sample).

Each of the databases provided the following metadata associated with the glycoprotein:

- UniCarbKB:
  $\circ $ Analytical techniques (glycomics and glycoproteomics), sample preparation/enrichment, disease, taxonomy, tissue, cell line, protein (peptide), glycan structure (composition), glycosylation site and abundance.
- GlyConnect
  $\circ $ Disease, tissue, taxonomy, cell line, protein variants, peptide, glycan structure (composition) and glycosylation site.
- GlycoNAVI
  $\circ $ Sample collection method, disease, taxonomy, cell line, protein, glycan composition, glycosylation site and glycan abundance ratio.

Thus, for the same glycoprotein, we attempted to find associated metadata outside the scope of GlycoCoO using SPARQL. We generated different SPARQL queries to integrate the data from these resources. Two examples are given below.

**Fig. 4:**
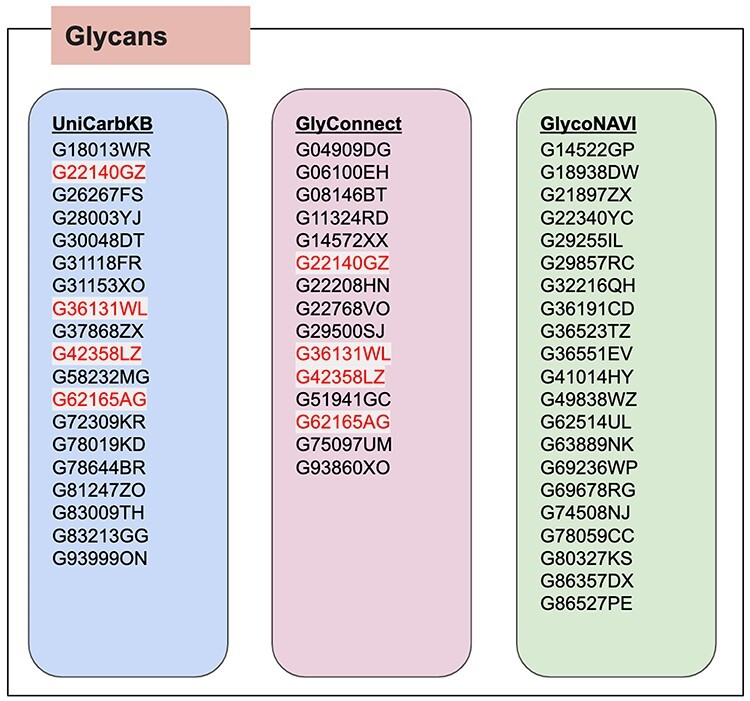
Result of searching the glycans attached to the same Haptoglobin protein (UniProt ID: P00738) from across three RDF-based glycoconjugate databases.

**Fig. 5:**
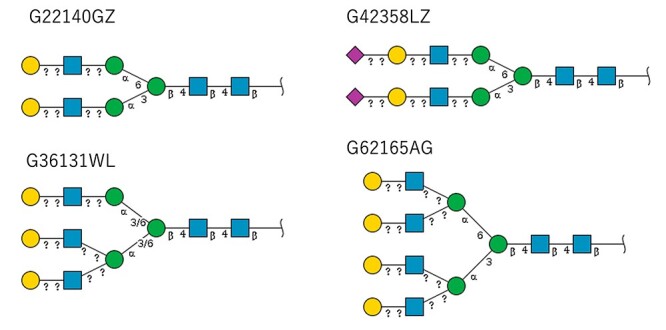
The four glycans found for the Haptoglobin glycoprotein that were common across all three databases.

First, a SPARQL query searching for the glycosylation sites on this protein was performed. The largest number of glycosylation sites (184, 207, 211, 241) were annotated in GlyConnect, while GlycoNAVI reported 184, 207, 211 and UniCarbKB reported 184, 187, 207, 211 and 241. Figure Fig. 4 illustrates the results of the SPARQL query used to find all glycans on this protein. For glycans, the red colored GlyTouCan IDs are those that were common (G22140GZ, G36131WL, G42358LZ and G62165AG) across two databases. Their images are shown in Figure Fig. 5. In the Supplementary Materials, we list the images for each glycan list from each respective database. From these images, it is clear that the glycans are fairly common across all databases; the only differences were the degrees of fractionation and ambiguities between glycans.

The following are the SPARQL queries that were used to obtain this data about glycosylation sites (query 1) and glycan structures (query 2) for haptoglobin.

### Example SPARQL query 1 (glycosylation sites)

prefix gco:<http://purl.jp/bio/12/glyco/conjugate#>

prefix dcterms:<http://purl.org/dc/terms/>

prefix faldo:<http://biohackathon.org/resource/faldo#>

prefix dcterms:<http://purl.org/dc/terms/>

select distinct ?g ?uniprot_id (str(?position) AS ?site)

where

{

{

graph ?g {

VALUES ?g { <http://glycoinfo.org/glycocoo/glyconnect> <http://glycoinfo.org/glycocoo/unicarbkb>}

# GlyConnect and UniCarbKB

?ref_conjugate gco:has_protein_part ?ref_protein.

?ref_protein gco:glycosylated_at ?region .

?region faldo:location ?location .

?location faldo:position ?position .

?ref_protein gco:has_protein ?protein .

?protein rdfs:seeAlso ?uniprot .

?uniprot dcterms:identifier ?uniprot_id .

}

}

UNION

{

# GlycoNAVI

SERVICE <https://sparql.glyconavi.org/sparql> {

graph ?g {

VALUES ?g { <http://glycoinfo.org/glycocoo/glyconavi> }

?ref_conjugate gco:has_protein_part ?ref_protein .

?ref_protein gco:glycosylated_at ?region .

?region faldo:location ?location .

?location faldo:position ?position .

?ref_protein gco:has_protein ?protein .

?protein rdfs:seeAlso ?uniprot .

?uniprot dcterms:identifier ?uniprot_id .

}

}

}

}

order by ?g ?position

### Example SPARQL query 2 (glycans)

# Glycan Part

prefix glycan:<http://purl.jp/bio/12/glyco/glycan#>

prefix gco:<http://purl.jp/bio/12/glyco/conjugate#>

prefix dcterms:<http://purl.org/dc/terms/>

prefix faldo:<http://biohackathon.org/resource/faldo#>

prefix sio:<http://semanticscience.org/resource/>

prefix foaf:<http://xmlns.com/foaf/0.1/>

select distinct ?g ?uniprot_id ?glytoucan_id

where

{

{

graph ?g{

VALUES ?g { <http://glycoinfo.org/glycocoo/glyconnect> <http://glycoinfo.org/glycocoo/unicarbkb>}

# GlyConnect & UniCarbKB

?glycoconjugate_ref gco:has_protein_part ?protein_part.

?protein_part gco:has_protein ?protein.

?protein rdfs:seeAlso ?uniprot.

?uniprot dcterms:identifier ?uniprot_id.

?glycoconjugate_ref gco:has_saccharide_part ?ref_sac.

?ref_sac glycan:has_glycan ?saccharide.

?saccharide foaf:primaryTopicOf ?glytoucan.

?glytoucan dcterms:identifier ?glytoucan_id.

}

}

UNION

{

# GlycoNAVI

SERVICE <https://sparql.glyconavi.org/sparql> {

graph ?g {

VALUES ?g{<http://glycoinfo.org/glycocoo/glyconavi>}

?glycoconjugate_ref gco:has_protein_part ?protein_part.

?protein_part gco:has_protein ?protein.

?protein rdfs:seeAlso ?uniprot.

?uniprot dcterms:identifier ?uniprot_id.

?glycoconjugate_ref gco:has_saccharide_part ?ref_sac.

?ref_sac glycan:has_glycan ?saccharide.

?saccharide foaf:primaryTopicOf ?glytoucan.

?glytoucan dcterms:identifier ?glytoucan_id.

}

}

}

}

order by ?g ?uniprot_id ?glytoucan_id

**Fig. 6:**
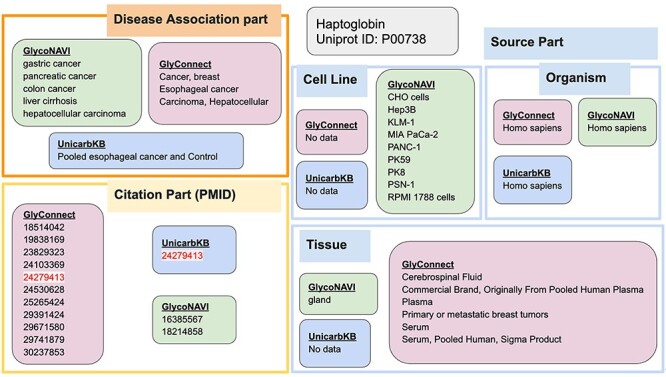
The disease annotations and relevant citations that were accumulated across the three databases for the same haptoglobin protein.

The next example illustrates the SPARQL query used to find all disease annotations, their citations, source and tissue information for this protein (Figure Fig. 6). Regarding Disease Associations, GlyConnect and UniCarbKB both reported *esophageal cancer*, while GlycoNAVI and GlyConnect both reported *hepatocellular carcinoma*. However, GlycoNAVI and GlyConnect both reported additional cancers that were not reported by any of the others. All three databases reported *Homo sapiens* as the organism, and only GlycoNAVI provides Cell Line information for this protein. Citations only overlapped between UniCarbKB and GlyConnect, most likely because both have data derived from GlycoSuiteDB. Finally, only GlyConnect contained data regarding Tissues.

The SPARQL queries to obtain disease (query 3), publication (query 4) and source information (query 5) are as follows.

### Example SPARQL Query 3 (Disease associations)

# Disease Association part

PREFIX glycan:<http://purl.jp/bio/12/glyco/glycan#>

PREFIX gco:<http://purl.jp/bio/12/glyco/conjugate#>

PREFIX skos:<http://www.w3.org/2008/05/skos#>

PREFIX dcterms:<http://purl.org/dc/terms/>

PREFIX faldo:<http://biohackathon.org/resource/faldo#>

PREFIX sio:<http://semanticscience.org/resource/>

PREFIX rdfs: <http://www.w3.org/2000/01/rdf-schema#>

SELECT DISTINCT ?g ?disease_label ?notation ?uniprot_id

WHERE

{

{

graph ?g {

VALUES ?g {<http://glycoinfo.org/glycocoo/glyconnect> <http://glycoinfo.org/glycocoo/unicarbkb>}

# GlyConnect & UniCarbKB

?glycoconjugate_ref glycan:has_association ?association;

gco:has_protein_part ?protein_part.

?association sio:SIO_000628 ?disease.

?disease rdfs:label ?disease_label.

?disease skos:notation ?notation.

?protein_part gco:has_protein ?protein .

?protein rdfs:seeAlso ?uniprot.

?uniprot dcterms:identifier ?uniprot_id.

VALUES ?uniprot_id {"P00738"}

}

}

UNION

{

# GlycoNAVI

SERVICE <https://sparql.glyconavi.org/sparql> {

graph ?g {

VALUES ?g {<http://glycoinfo.org/glycocoo/glyconavi>}

?glycoconjugate_ref glycan:has_association ?association;

gco:has_protein_part ?protein_part.

?association sio:SIO_000628 ?disease.

?disease rdfs:label ?disease_label.

?disease skos:notation ?notation.

?protein_part gco:has_protein ?protein.

?protein rdfs:seeAlso ?uniprot.

?uniprot dcterms:identifier ?uniprot_id.

VALUES ?uniprot_id {"P00738"}

}

}

}

}

ORDER BY ?g

### Example SPARQL Query 4 (Publications)

prefix glycan:<http://purl.jp/bio/12/glyco/glycan#>

prefix gco:<http://purl.jp/bio/12/glyco/conjugate#>

prefix dcterms:<http://purl.org/dc/terms/>

prefix rdfs: <http://www.w3.org/2000/01/rdf-schema#>

select distinct ?g ?uniprot_id (str (?pmid) AS ?PMID)

where

{

{

graph ?g {

VALUES ?g { <http://glycoinfo.org/glycocoo/glyconnect> <http://glycoinfo.org/glycocoo/unicarbkb>}

?glycoconjugate_ref glycan:published_in ?citation.

?glycoconjugate_ref gco:has_protein_part ?protein_part.

?protein_part gco:has_protein ?protein.

?protein rdfs:seeAlso ?uniprot.

?uniprot dcterms:identifier ?uniprot_id.

VALUES ?uniprot_id {"P00738"}

# glyconnect & unicarbkb

?citation dcterms:references ?pubmed.

?citation glycan:has_pmid ?pmid.

}

}

UNION

{

# GlycoNAVI

SERVICE <https://sparql.glyconavi.org/sparql> {

graph ?g {

VALUES ?g {<http://glycoinfo.org/glycocoo/glyconavi>}

?glycoconjugate_ref glycan:published_in ?citation.

?glycoconjugate_ref gco:has_protein_part ?protein_part.

?protein_part gco:has_protein ?protein.

?protein rdfs:seeAlso ?uniprot.

?uniprot dcterms:identifier ?uniprot_id.

VALUES ?uniprot_id {"P00738"}

?citation dcterms:references ?pubmed.

?citation glycan:has_pmid ?pmid.

}

}

}

}

order by ?g ?pmid

Example SPARQL Query 5 (Biological source associations)

prefix glycan:<http://purl.jp/bio/12/glyco/glycan#>

prefix gco:<http://purl.jp/bio/12/glyco/conjugate#>

prefix dcterms:<http://purl.org/dc/terms/>

prefix faldo:<http://biohackathon.org/resource/faldo#>

prefix sio:<http://semanticscience.org/resource/>

prefix dcterms:<http://purl.org/dc/terms/>

prefix up: <http://purl.uniprot.org/core/>

select distinct ?g ?uniprot_id ?tissue ?cell_line ?organism

where

{

{

graph ?g {

VALUES ?g {<http://glycoinfo.org/glycocoo/glyconnect> <http://glycoinfo.org/glycocoo/unicarbkb>}

# glyconnect & unicarbkb

?glycoconjugate_ref glycan:is_from_source ?source; gco:has_protein_part ?protein_part.

optional {?source glycan:has_tissue ?tissue.}

optional {?source glycan:has_cell_line ?cell_line.}

?protein_part gco:has_protein ?protein.

?protein rdfs:seeAlso ?uniprot.

?uniprot dcterms:identifier ?uniprot_id.

VALUES ?uniprot_id {"P00738"}

optional {

?source glycan:has_taxon ?taxon.

OPTIONAL {?taxon up:scientificName ?organism.}

}

}

}

UNION

{

# GlycoNAVI

SERVICE <https://sparql.glyconavi.org/sparql> {

graph ?g {

VALUES ?g {<http://glycoinfo.org/glycocoo/glyconavi>}

?glycoconjugate_ref glycan:is_from_source ?source;

gco:has_protein_part ?protein_part.

optional{?source glycan:has_tissue ?tissue.}

optional{?source glycan:has_cell_line ?cell_line.}

optional{

?source glycan:has_taxon ?taxon.

optional {?taxon up:scientificName ?organism.}

}

?ref_conjugate gco:has_protein_part ?ref_protein.

?ref_protein gco:has_protein ?protein .

?protein rdfs:seeAlso ?uniprot.

?uniprot dcterms:identifier ?uniprot_id.

VALUES ?uniprot_id {"P00738"}

}

}

}

}

order by ?g ?tissue ?cell_line ?taxon

## Discussion

GlycoCoO is a novel compact ontology for describing protein and lipid glycosylation in a consistent manner that can be easily adopted by the broader omics community. It is a dynamic ontology that can be used to describe known glycosylation features, site-specific glycoforms, abundance data, and where available descriptions of experimental conditions and methods. It is available in BioPortal at https://bioportal.bioontology.org/ontologies/GLYCOCOO as well as on GitHub https://github.com/glycoinfo/GlycoCoO where the Wiki page illustrates examples of usage and provides the RDF data described in this manuscript.

As illustrated with the SPARQL queries described in the Results, multiple databases could be queried using a single query to retrieve integrated information regarding a single glycoprotein. Diverse information ranging from disease associations to tissues and cell lines could be retrieved from a large number of publications. We note that all of these databases are continuously being updated, therefore, the current data is only a reflection of the data at the time of this writing. Regarding the glycan data, as shown in Supplementary Materials, it is evident that although the GlyTouCan IDs did not overlap, the IDs that were assigned could be mapped to other glycans due to differences in fragmentation annotations and ambiguous linkages. GlyTouCan provides relationship information regarding such ambiguities, and further analysis of these glycan relationships are left for future work.

In this work, we have provided examples of the RDF data for glycoproteins that have been developed by GlycoNAVI, GlyConnect and UniCarbKB. Another resource that provides glycoprotein information in RDF form is GlyGen (https://glygen.org), which is adopting GlycoCoO concepts to support data interoperability. We are also planning on contacting lipid ontology and database developers to discuss where concepts could be combined or mapped with one another. Eventually, all of these integrated data will be available from the members of the GlySpace Alliance (Aoki-Kinoshita et al. 2020).

Moreover, having shown the effectiveness of the GlycoCoO ontology, we will survey ways to integrate with existing related ontologies. For example, the Protein Ontology (PRO) provides a robust and scalable ontological research infrastructure (Natale et al. 2011) for proteins. It serves as a standardized representation of proteoforms using UniProtKB as a sequence reference and PSI-MOD as a post-translational modification reference to richly and accurately model protein entities and their relationships in biological systems. As part of the GlyGen initiative PRO will be expanded to capture the complexity of glycoproteoforms, in particular the heterogeneity of site-specific protein glycosylation, by aligning with the GlycoCoO concepts described.

With these developments of ontologies and databases based on an agreed standard for glycoconjugates, a large proportion of life science data can be integrated. However, this will require the adoption of these standards by all parties involved, which may entail much promotion and discussion with various communities. Eventually, GlycoCoO can serve as the basis of a glycoconjugate repository, whereby accession numbers can be assigned to such molecules.

## Supplementary Material

GlycoCoO_Supp_cwab013
