# Corrigendum to: The glycoconjugate ontology (GlycoCoO) for standardizing the annotation of glycoconjugate data and its application

**DOI:** 10.1093/glycob/cwab065

**Published:** 2021-08-10

**Authors:** Issaku Yamada, Matthew P Campbell, Nathan Edwards, Leyla Jael Castro, Frederique Lisacek, Julien Mariethoz, Tamiko Ono, Rene Ranzinger, Daisuke Shinmachi, Kiyoko F Aoki-Kinoshita

**Affiliations:** Research Department, The Noguchi Institute, 1-9-7 Kaga, Itabashi, Tokyo 173-0003, Japan; Institute for Glycomics, Griffith University at Gold Coast, Southport, QLD 4215, Australia; Department of Biochemistry, Molecular and Cellular Biology, Georgetown University Medical Center, Washington, D.C. 20007, USA; ZB MED Information Centre for Life Sciences, Gleueler Str. 60, 50931 Cologne, Germany; Proteome Informatics Group, SIB Swiss Institute of Bioinformatics, Computer Science Department, University of Geneva, route de Drize 7, CH - 1227 Geneva Switzerland, and also Section of Biology, University of Geneva, Geneva, Switzerland; Proteome Informatics Group, SIB Swiss Institute of Bioinformatics, 7 Route de Drize, 1227 Geneva, Switzerland; Faculty of Science and Engineering, Soka University, 1-236 Tangi-machi, Hachioji, Tokyo 192-8577, Japan; Complex Carbohydrate Research Center, The University of Georgia, 315 Riverbend Rd, Athens, Georgia 30602, USA; R&D Department, SparqLite LLC., 1615-22 Ishikawamachi, Hachioji, Tokyo 192-0032, Japan; Glycan & Life Science Integration Center (GaLSIC), Faculty of Science and Engineering, Soka University, 1-236 Tangi-machi, Hachioji, Tokyo 192-8577, Japan

*Glycobiology*, cwab013, doi: https://doi.org/10.1093/glycob/cwab013.

Issaku Yamada^1,2^, Matthew P. Campbell^3^, Nathan Edwards^3^, Leyla Jael Castro^4^, Frederique Lisacek^5^, Julien Mariethoz^6^, Tamiko Ono^7^, Rene Ranzinger^8^, Daisuke Shinmachi^9^ and Kiyoko F. Aoki-Kinoshita^10^

^2^Research Depertment, The Noguchi Institute, 1-9-7 Kaga, Itabashi, Tokyo 173-0003, Japan, ^3^Institute for Glycomics, Griffith University at Gold Coast, Southport, QLD 4215, Australia, ^4^Department of Biochemistry, Molecular and Cellular Biology, Georgetown University Medical Center, Washington, D.C. 20007, USA, ^5^ZB MED Information Centre for Life Sciences, Gleueler Str. 60, 50931 Cologne, Germany, ^6^Proteome Informatics Group, SIB Swiss Institute of Bioinformatics, Computer Science Department, University of Geneva, route de Drize 7, CH – 1227 Geneva Switzerland, and also Section of Biology, University of Geneva, Geneva, Switzerland, ^7^Proteome Informatics Group, SIB Swiss Institute of Bio informatics, 7 Route de Drize, 1227 Geneva, Switzerland, ^8^Faculty of Science and Engineering, Soka University, 1-236 Tangi-machi, Hachioji, Tokyo 192-8577, Japan, ^9^Complex Carbohydrate Research Center, The University of Georgia, 315 Riverbend Rd, Athens, Georgia 30602, USA, ^10^R&D Department, SparqLite LLC., 1615-22 Ishikawamachi, Hachioji, Tokyo 192-0032, Japan, and ^11^Glycan & Life Science Integration Center (GaLSIC), Faculty of Science and Engineering, Soka University, 1-236 Tangi-machi, Hachioji, Tokyo 192-8577, Japan

^1^To whom correspondence should be addressed: Tel: +81-3-5944-3214; Fax: +81-3-3961-4071; e-mail: issaku@noguchi.or.jp.

They should have read:

Issaku Yamada^1,2^, Matthew P. Campbell^3^, Nathan Edwards^4^, Leyla Jael Castro^5^, Frederique Lisacek^6^, Julien Mariethoz^7^, Tamiko Ono^8^, Rene Ranzinger^9^, Daisuke Shinmachi^10^ and Kiyoko F. Aoki-Kinoshita^11^

^2^Research Depertment, The Noguchi Institute, 1-9-7 Kaga, Itabashi, Tokyo 173-0003, Japan, ^3^Institute for Glycomics, Griffith University at Gold Coast, Southport, QLD 4215, Australia, ^4^Department of Biochemistry, Molecular and Cellular Biology, Georgetown University Medical Center, Washington, D.C. 20007, USA, ^5^ZB MED Information Centre for Life Sciences, Gleueler Str. 60, 50931 Cologne, Germany, ^6^Proteome Informatics Group, SIB Swiss Institute of Bioinformatics, Computer Science Department, University of Geneva, route de Drize 7, CH – 1227 Geneva Switzerland, and also Section of Biology, University of Geneva, Geneva, Switzerland, ^7^Proteome Informatics Group, SIB Swiss Institute of Bioinformatics, 7 Route de Drize, 1227 Geneva, Switzerland, ^8^Faculty of Science and Engineering, Soka University, 1-236 Tangi-machi, Hachioji, Tokyo 192-8577, Japan, ^9^Complex Carbohydrate Research Center, The University of Georgia, 315 Riverbend Rd, Athens, Georgia 30602, USA, ^10^R&D Department, SparqLite LLC., 1615-22 Ishikawamachi, Hachioji, Tokyo 192-0032, Japan, and ^11^Glycan & Life Science Integration Center (GaLSIC), Faculty of Science and Engineering, Soka University, 1-236 Tangi-machi, Hachioji, Tokyo 192-8577, Japan

^1^To whom correspondence should be addressed: Tel: +81-3-5944-3214; Fax: +81-3-3961-4071; e-mail: issaku@noguchi.or.jp.

The paper has now been corrected online.

