# Supplementary material for: The glycoconjugate ontology (GlycoCoO) for standardizing the annotation of glycoconjugate data and its application
Source: Glycobiology. 2021 Feb 23;31(7):741–50. doi: 10.1093/glycob/cwab013 (PMC8351504; doi:10.1093/glycob/cwab013)
Supplement: GlycoCoO_Supp_cwab013 [file glycocoo_supp_cwab013.pdf]

Title:
The Glycoconjugate Ontology GlycoCoO for standardizing the annotation of glycoconjugate data and its application

Supplementary Materials

- Figures S1 UniCarbKB Glycans
- Figures S2 Glyconnect Glycans
- Figures S3 GlycoNAVI Glycans

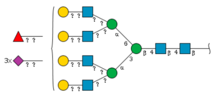

GlyTouCan-SNFG:G18013WR

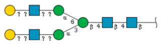

GlyTouCan-SNFG:G22140GZ

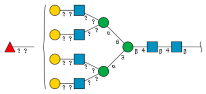

GlyTouCan-SNFG:G26267FS

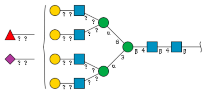

GlyTouCan-SNFG:G28003YJ

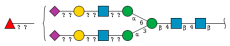

GlyTouCan-SNFG:G30048DT

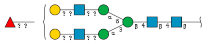

GlyTouCan-SNFG:G31118FR

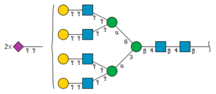

GlyTouCan-SNFG:G31153XO

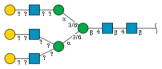

GlyTouCan-SNFG:G36131WL

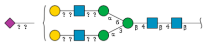

GlyTouCan-SNFG:G37868ZX

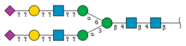

GlyTouCan-SNFG:G42358LZ

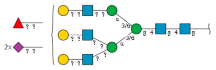

GlyTouCan-SNFG:G58232MG

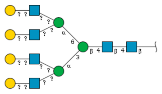

GlyTouCan-SNFG:G62165AG

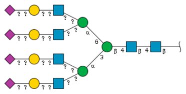

GlyTouCan-SNFG:G72309KR

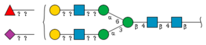

GlyTouCan-SNFG:G78019KD

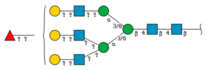

GlyTouCan-SNFG:G78644BR

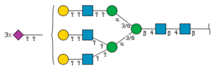

GlyTouCan-SNFG:G81247ZO

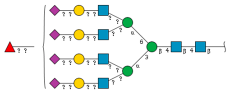

GlyTouCan-SNFG:G83009TH

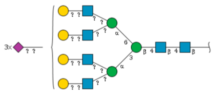

GlyTouCan-SNFG:G83213GG

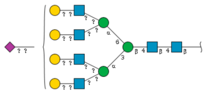

GlyTouCan-SNFG:G93999ON

Figures S1 UniCarbKB Glycans

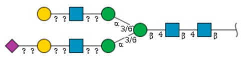

GlyTouCan-SNFG:G04909DG

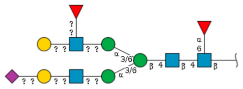

GlyTouCan-SNFG:G06100EH

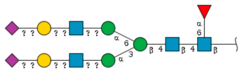

GlyTouCan-SNFG:G08146BT

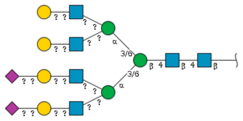

GlyTouCan-SNFG:G11324RD

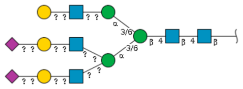

GlyTouCan-SNFG:G14572XX

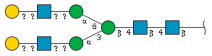

GlyTouCan-SNFG:G22140GZ

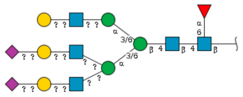

GlyTouCan-SNFG:G22208HN

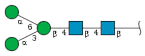

GlyTouCan-SNFG:G22768VO

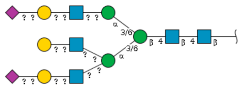

GlyTouCan-SNFG:G29500SJ

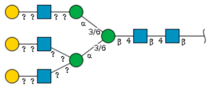

GlyTouCan-SNFG:G36131WL

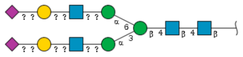

GlyTouCan-SNFG:G42358LZ

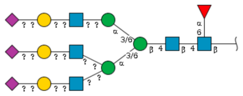

GlyTouCan-SNFG:G51941GC

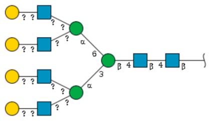

GlyTouCan-SNFG:G62165AG

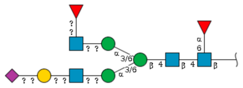

GlyTouCan-SNFG:G75097UM

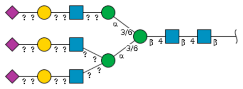

GlyTouCan-SNFG:G93860XO

Figures S2 Glyconnect Glycans

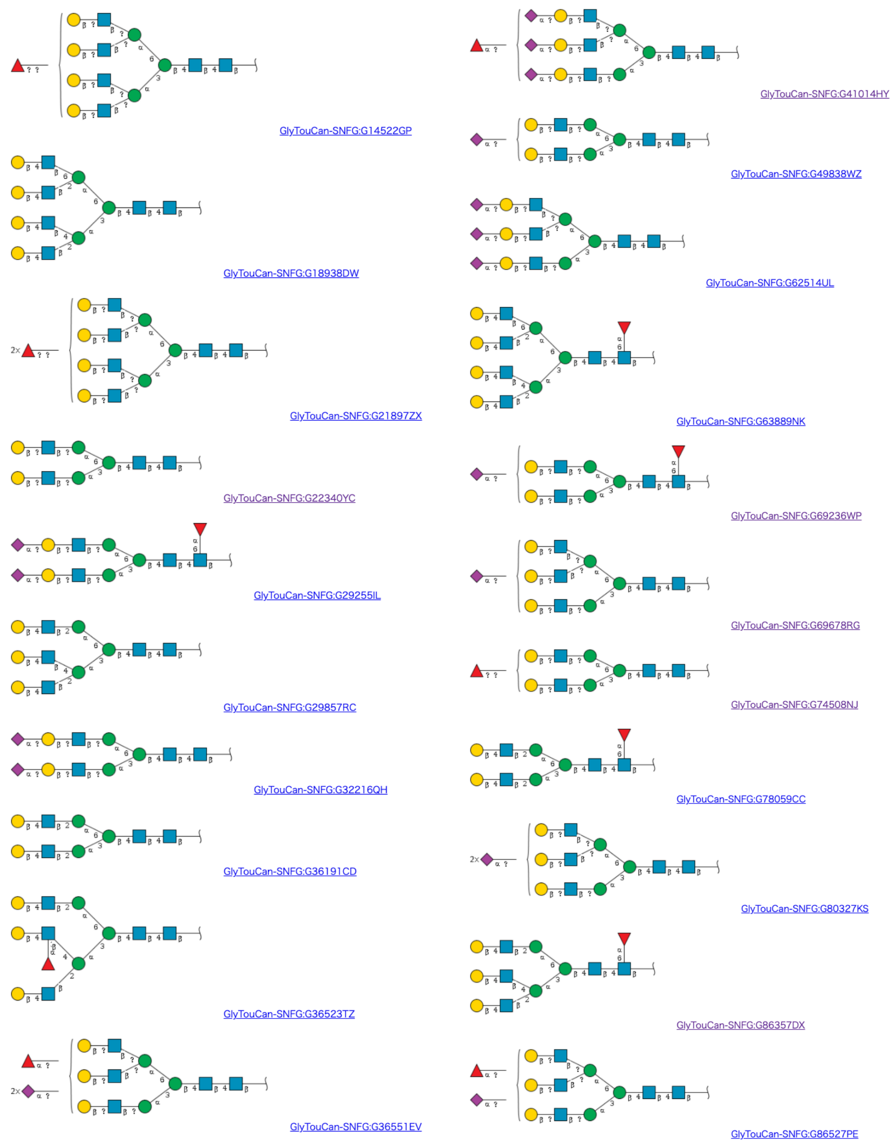

Figures S3 GlycoNAVI Glycans
